# Characteristics of the skills of caregivers of people with dementia: observational study

**DOI:** 10.1186/s12875-020-01218-6

**Published:** 2020-07-25

**Authors:** Marie-Conception Leocadie, Jean-Manuel Morvillers, Sophie Pautex, Monique Rothan-Tondeur

**Affiliations:** 1 HES-SO University of Applied Sciences and Arts of Western Switzerland, School of Health Sciences, Geneva, Switzerland; 2 Université Sorbonne Paris Nord, Chaire Recherche Sciences infirmières, Laboratoire Educations et Pratiques de Santé (LEPS), (EA 3412), UFR SMBH, F-93017 Bobigny, France; 3 GHU Paris Psychiatrie et Neurosciences, Hôpital Sainte Anne, Paris, France; 4 Geriatric Unit and Community Palliative Care, Geneva University Hospitals, Geneva, Switzerland; 5 AP HP, Nursing Sciences Research chair, Paris, France

**Keywords:** Caregivers, Alzheimer’s, Dementia, Skills, Observational study

## Abstract

**Background:**

Due to demographic change within an aging population as announced by the WHO, the involvement of caregivers is essential. Caregivers are required to change their roles within the family unit. Such life transitions experienced by caregivers to people confronted with dementia-type pathologies are sometimes difficult, necessitating the acquisition and development of certain skills. Few studies have shown that caregivers develop specific and essential skills to promote quality care and safety. To characterize their skills, there is a need to identify the abilities, knowledge, resources, obstacles and constraints that contribute to caregivers’ transitions. The research question for this study was: What skills do caregivers use to care for their loved one with dementia?

**Methods:**

Qualitative observational research based on the epistemological paradigm of socioconstructivist knowledge was conducted. The study was carried out in the canton of Geneva and recruitment was carried out through the participation of the Alzheimer’s association and the association for the support and assistance of elderly people in medical and social institutions and their families (APAF). Observations and semi-structured interviews were conducted in the homes of 14 family carers caring for their loved one with dementia. The observations were transcribed on observation grids and the interviews were recorded. Subsequently, according to the classic distinction of Denzin (Interpretive interractionism, 2001), we analysed the observation notes and verbatims, then as recommended by Miles et al. (Qualitative data analysis: a methods sourcebook, 2014), two researchers triangulated the results.

**Results:**

The results identified five types of situations regularly experienced by caregivers. The study characterized 11 skills that caregivers use to cope with their daily lives. The learning process and maladaptive behaviours in caring for their loved ones with dementia were also highlighted.

**Conclusion:**

This study was able to point out that today’s caregivers have developed more competency than their predecessors. This evolution can be explained by new paradigms of care requiring caregivers to be more involved. Although some caregivers need training, others through their experiences can act upon and provide knowledge. To improve the quality and safety of care for people with dementia, this expertise can be the subject of partnerships between caregivers and health care staff.

## Background

In the context of demographic change with an aging population announced by the WHO, the involvement of caregivers is essential [1]. Dementia is one of the most common diseases in the elderly. Nearly 35.6 million people worldwide have dementia, a number that is expected to double by 2030 (to 65.7 million) and more than triple by 2050 (to 115.4 million) [2]. In Switzerland, the typology of family carers is almost similar to that identified in other European countries; we find between 74 and 82% female carers. They are essentially spouses and children, 71% of whom live in the same home as the sick person. On the other hand, the average age of Swiss carers is slightly higher than in Europe (65.6 years). Spouses, who make up 51% of the caregiver population, are on average 77 years old, while children, who make up 49% of caregivers, are on average 57 years old. Approximately half care for spouses and half for parents. Individuals are primarily cared for by close family members, with siblings and stepchildren rarely involved. However, if help is provided, it is daughters-in-law who care for their sick in-laws [3].

A caregiver is defined as “a person in the immediate circle of an individual who is dependent on assistance with certain activities of daily living, who, on a non-professional and informal basis, provides him/her with regular support services of care or presence, of a varied nature and intensity designed to compensate disabilities, difficulties, ensure security, maintenance identity and social bond. Caregivers can be family members, neighbours or friends. This does not concern organized forms of volunteering” [4].

The status of caregiver requires a lifestyle change in terms of role, organization, life plans etc., which we characterize as a “life transition” [5]. The transition of life experienced by caregivers of people confronted with dementia is sometimes difficult. Caregivers of people with dementia are very stressed and busy every day, with physical, psychological, social and financial consequences [6–11]. These repercussions often entail “feeling of burden”, a phenomenon first described by Zarit et al. in 1986 [12]. A quantitative study has highlighted correlations between the multiple tasks performed by caregivers and the appearance over time of a feeling called “burden” [10]. The feeling of burden is defined as “all the physical, psychological, emotional, social and financial consequences borne by the caregivers” [13].

The majority of the research conducted to date has been based on the effects of caring on family caregivers, assessing their needs and the effects of the interventions put in place to support them [14–17]. As the role of caregiving demands accompanying someone in a complex way that generates responsibilities, this new status engages caregivers in a learning process that constantly requires them to acquire new skills [18]. To support them in the acquisition of skills, several intervention programmes have emerged. A systematic review led by the Cheng, S.T. research team published in 2019 and 2020 concluded that “Educational programs with psychotherapeutic components, counseling/psychotherapy, and mindfulness-based interventions have the strongest effects on reducing depressive symptoms. Multicomponent and miscellaneous interventions have the largest effects on reduction of burden/stress. Multicomponent and mindfulness-based interventions have the largest effects on enhancing subjective well-being” [19, 20]. Previous studies such as REACH and multiple meta-analyses confirmed these results, indicating that family caregivers need multi-component and tailored interventions [11, 21–23].

Nevertheless, in the absence of help, caregivers must develop skills to cope with daily life and avoid the feeling of burden. The notion of skill development is important to enable the process of transition to the role of caregiver. To master unfamiliar situations, knowledge allows caregivers to develop adaptability and self-confidence [5]. The expertise developed by family/informal caregivers helps them make decisions and assert their role. Regarding the skills of caregivers, very few studies have shown that caregivers develop specific and essential skills over the years to support and promote quality care to their loved ones [18, 24]. The studies carried out so far have highlighted the actions undertaken by caregivers but have not described the elements that make it possible to build the necessary skills. Several questions remain: on which knowledge and processes are caregivers’ skills built? What are the elements they rely on to develop their skills?

Answers to these questions are important and can be tools for people who accompany caregivers going through transitions. This knowledge is also important for academic, clinical and training purposes.

Indeed, as Jonnaert described, building skills is based on one’s ability to handle a situation. Caregivers’ skills are based on their abilities, knowledge, resources, obstacles, constraints and reflections. As defined by Jonnaert P. in 2009 “through a skill, a subject mobilizes, selects and coordinates a series of resources (including some of his knowledge, but a series of other resources that would be affective, social and those related to the situation and its constraints) to deal effectively with a situation. Competence implies, beyond effective treatment, that the same subject takes a critical look at the results of this treatment, which must be socially acceptable” Concerning the application of a competence, Jonnaert mentioned in his research, a cascade approach. “According to the situations in which a skill mobilizes them, the abilities in turn mobilize this or that skill” which in turn calls for disciplinary content [25]. Jonnaert evokes a cascade approach to mobilization [26, 27].

To identify and characterize the skills of family/informal caregivers, an observational study which identifies and characterizes the skills of caregivers of people with dementia, was considered.

The aim of this study was to not only describe the skills and processus that caregivers have developed.

## Methods

This study represents observational qualitative research based on the epistemological paradigm of knowledge of socioconstructivism [26, 27].

### Setting

We conducted the study in the community of Geneva, Switzerland in 2017 and 2018. The study took place directly in the homes of caregivers for the sake of observing their real lives and increasing their availability. In the same way, most of the interviews took place at home, only two being conducted at the Geneva School of Health as requested by the caregivers.

### Participants

Eligible participants met the following inclusion criteria:

- being a caregiver of any type (spouse, child, neighbour, friend etc.) who meets the definition of “caregiver” formulated by the General Directorate of Health in 2012;
- being a caregiver to someone with dementia-like disorders with any degree of impairment;
- having accompanied a family member for 2 years minimum, thus demonstrating experience as a caregiver; and
- being fluent in French.

Recruitment was carried out through the participation of the Alzheimer’s Association, the APAF (Association for Assistance and Support for the Elderly in Social Medical Establishments and their families) and the Imad (Institute Geneva Home Support). Two caregivers were recruited via the snowball method.

The inclusion criteria did not require that caregivers and their family member with dementia reside in the same household.

### Data collection

#### Observations

The research investigator made observations in different situations in the presence of their loved one with dementia. (Situations related to daily life such as exchanges with the health network, negotiation with the sick family member in the performance of daily tasks, the daily organisation of the family member confronted with dementia, communication, actions to regulate crises related to the illness etc.), which are defined as “a data collection tool where the researcher becomes a witness of the behaviours of the individuals and the practices within the groups staying at the same places where they are taking place” [28]. Observations took place over the course of a day at the caregivers’ homes. The objective was to be able to observe the skills used by the caregivers during daily activities with their family members. After the observations, all the grids and manual notes were transcribed using Word software (observation grid constructed and completed as observations are made. The observation grid was considered complete when the identified themes had reached saturation). Martineau [28] recommended all of these research actions.

#### Interviews

We based the semi-directed interviews on general areas related to skills implemented by the caregivers during different experiences lived on a daily basis. The purpose of the interviews was to explain or clarify certain events or facts that the caregivers observed or experienced. To guide the interviews, we asked some questions corresponding to the situations we observed in view of particular activities of the participants.

The aim of these interviews was to verbalize treatment of previously observed situations, discuss goals to be attained, make sense of caregivers’ actions and identify the knowledge on which their actions were based. We recorded the interviews anonymously using a tape recorder.

### Data analysis

At first, we analysed the observation notes and verbatim transcripts in a singular way; that is, we analysed each document of notes and each recorded interview according to the theoretical framework described by Jonnaert. The principal investigator codified all activities that the caregivers performed and categorized them to identify what skills, knowledge, abilities, resources and constraints were used and met by caregivers when caring for their loved ones in the face of disease.

From this first analysis, we carried out a second one based on the combination of previous elements to identify the skills and situations to which they applied. To proceed with the coding, once they were transcribed, we transferred the verbatim interviews and observation notes to MAXQDA12 software.

We coded the data in three stages. During the first coding cycle, according to Jonnaert’s theory, we carried out a deductive analysis classifying verbatim statements as representative of abilities (know-how or knowing how to be), knowledge, resources, constraints, obstacles and reflections of caregivers. This first step corresponded to the recommendations of several authors to assign codes to verbatim statements that symbolically represent descriptive or deductive information. These codes were attributed to large parts of the participants’ statements; they favoured grouping segments of data represented by words or short sentences [25, 29].

The second coding cycle made it possible to classify the codes into themes. These themes corresponded to the different elements that made up a skill as described by Jonnaert. According to Miles et al. [30], themes represent pattern codes, which are considered to group these summaries into smaller numbers of topics.

We then categorized the different themes (skills), represented by the types of situations in which different competencies were mobilized. According to Denzin’s classic distinction, the principal investigator and research assistant attached to the project validated the analysis of all the interviews and observations [31]. On the basis of the various documents available (verbatim interviews, observation notes and time notes of the caregivers’ activities), cross-checking allowed for a non-biased validation [30]. The principal investigator and research assistant checked for the presence of different themes in the interviews and observation notes and discussed misunderstandings to facilitate closer analysis of participants’ statements and observations. After negotiation and validation of the final model, the principal investigator made a literary analysis based on the results of the Word software.

### Ethical considerations

We considered ethical aspects as part of this study. The research protocol was approved by the ethical commission legitimized for this purpose. Concepts related to the modalities of observations and interviews, the option of withdrawal for participants, and a guarantee of participant anonymity were stipulated and sent in writing. During the observations and interviews, we requested written consent signed by the caregivers.

## Results

Fourteen family caregivers aged 37 to 82 were included in the study. Of the participants, 7 were women and 7 were men. Nine were spouses, 3 were children and 2 were parents. For one of the participants, the family member being cared for is now deceased. One participant is in professional practice, 2 are unemployed and 11 are retired. The study gave rise to 12 observations (2 parents who form a couple and who were observed at the same time) and 14 interviews (see Table 1). No family caregiver has benefited from a specific intervention to support them. However, the majority were members of the Alzheimer’s association.

**Table 1:** Characteristics participants

**Characteristics of participants interviewed and observed**	**Number of years (*N* total = 14)**
**Gender type**	
Femme	7
Homme	7
**Socioeconomic status**	
Retired	11
Employee	1
Unemployed	2
**Family connection**	
Spousals	9
Parents	2
Child	3
**Number of years of support**	
Number of years of spousals support	7 (+/− 3.28)
Number of years of childs support	4
Number of years of parents support	5
**Age group**	
Under 40 years old	1
41–60 years old	2
61–80 years old	9
81 years old and over	2

Analysis of the combined data from the interviews and observation notes identified everyday situations that encourage learning and application of one or more skills. These situations contribute to the developmental processes necessary for many smooth transitions.

Elements that enabled the construction of skills were highlighted. In addition, data analysis highlighted the behaviours of caregivers who did not achieve the results initially sought. In each following section, verbatim reports are presented (black for the interviews and blue for the observation notes).

## Situations and skills

**Fig. 1:**
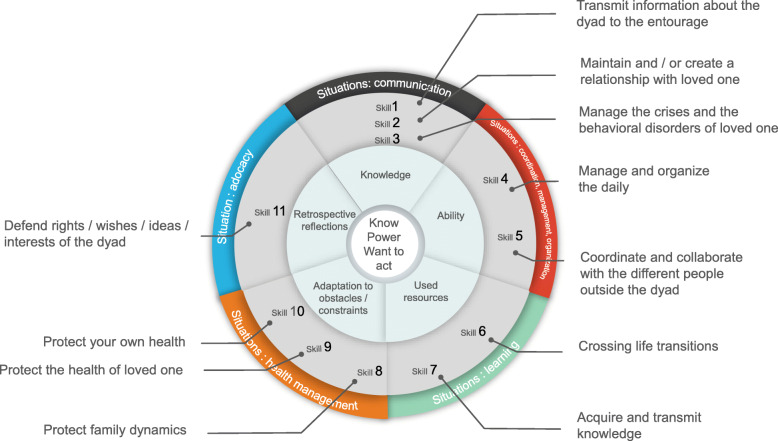
Caregivers competencies

We studied five types of situations that represent daily contexts caregivers can deal with. Managing each situation requires the combination of several skills. We also identified 11 abilities (see Fig. 1).

Through the application of skills, the study has highlighted the process of combining the capacities convened, which consist of knowledge, skills, resources, barriers, constraints and reflections of caregivers on their actions. In this article, we will only explain the data related to the skills; the other data will be described in a second article.

### Abilities

#### Transmit information about the dyad to the entourage

This skill is based on conveying relevant information to a person’s entourage concerning a loved one’s life story, health and future. In this study, the term entourage refers to health professionals, family and friends. Caregivers transmit information based on their own characteristics (cognitive abilities, traits, food preferences etc.) in a structured and chronological manner. To transmit information to an entourage, caregivers are transparent and deliver the story of the loved one without harming them.

- ***“ ... uh, I ... I lift the weight. This morning she was 65.3 (...)***
- ***Good, everything is noted ...***
- ***Yes, that’s what I see, yes.”***

#### Maintain and/or create a relationship with loved ones

Caregivers maintain relationships by creating moments of complicity around common or future activities. These relationships are based on stories of living together with the loved one and on respect given to the person facing the disease. When caregivers tell such stories, they solicit their loved ones during the conversation and thereby make them participate in the decision-making process. Caregivers try to find meaning in their loved ones’ speech, behaviour and questions, even if they are not always intelligible. Some caregivers can go so far as to create a virtual reality based on an unintelligible conversation that does not make sense. The creation of such a relationship requires great patience and respect and is based on both exchanges that evoke the caregiver’s love and a participative attitude that stimulates the person facing the illness.

***Observation note (ON): The caregiver helps her mother talk, looks at her, and explains her daily life.***

***ON: The caregiver tries to understand the actions of her mother and to find meaning (attitude of understanding).***

#### Manage the crises and behavioural disorders of close friends

During a crisis, after going through multiple explanations, negotiations and demonstrations, caregivers do not insist and avoid confrontation. They choose not to aggravate situations and take a step back. Caregivers voluntarily stop a conversation by, for example, changing rooms. To avoid a crisis, caregivers seek to understand the behaviour and reactions of their loved ones with regard to dementia. By observing these people’s behaviour, caregivers can identify any source of anxiety, which can help them prevent a situation. They try to control crises by anticipating day-to-day activities; however, a crisis is unavoidable when a loved one with dementia finds himself/herself in a new context. Caregivers in such cases adapt to the needs and desires of their loved ones, taking the time to calmly correct them and reorient their attention by either changing the subject or using humour. Some caregivers choose not to correct their loved ones, in keeping with preserving the dignity of the ill. This means that caregivers must adapt their behaviour towards their loved ones, which can in turn require integrating possible hallucinations during their interactions. Sometimes, depending on the personality of the loved one, caregivers may raise their voice, use a firmer tone or calmly suggest an idea without any aggressiveness.

Nevertheless, some caregivers may sometimes react differently without realizing it. When under pressure, faced with confrontation, provocation, guilt or deprecation, they may become angry and raise their voice.

To succeed in controlling behavioural disorders of a loved one, caregivers are constantly controlling their own emotions. They adopt attitudes to establish serenity within the dyad. To do this, they remain calm, show patience, play down conflicts and demonstrate a passive attitude or resignation. Caregivers might be active but are actually in a form of meditation.

- ***“ I can’t do it anymore.***
- ***Yeah, what do you do when it gets to be too much? When you are fed up?***
- ***Either I go to my office or I’m doing stuff, uh ... or I play or ... I do things that I have to do, and this helps.***
- ***This helps?”***

#### Manage and organize the daily routines

In order to organize different daily tasks, caregivers adopt several strategies. They are careful not to waste time, prioritizing tasks and/or doing several things at once. They constantly anticipate things to better manage their daily lives. To do this, they use a number of tools (paper or electronic diary, table with specific colours for each activity or family member etc.). They carefully organize activities so as not to provoke crises or accidents and use the same rituals every day with regard to activities and tasks. They perform these activities and tasks while constantly keeping an eye on their loved ones to ensure their safety. They adopt strategies based on the pathological behaviour of their loved ones (e.g., by making important object retention or arranging things strategically to find them). On a day-to-day basis, they help with shopping, housework, cooking and leisure activities, taking into account the constraints of the loved one’s illness. This daily organization asks caregivers to adapt, be rigorous, not procrastinate and, above all, be indulgent with themselves. Although the caregiver strives for perfection, it often remains unattainable.

***ON: Mr X arranges things so that he is close to his wife, which allows him to watch her and keep an eye on her (organization oriented around the safety of sick relatives).***

***ON: The activities of the day are ritualized. The loved one seems to be doing things by automatism without major problems. The caregiver additionally describes the activities and emphasizes a certain ritualization, especially concerning morning activities.***

#### Coordinate and collaborate with different people outside the dyad

Several types of actions are related to coordination and collaboration. Caregivers coordinate and collaborate with health, home and work staff. Coordination is also present within families and during tasks related to administrative management.

For collaboration to be effective, family caregivers must be transparent and honest in their dealings with staff and families, knowing how to position themselves and how to be firm and demanding. However, caregivers also exhibit behaviours such as negotiation, collegiality and self-improvement. This skill requires being attentive to the needs of a loved one, able to adapt, organized, perseverant and responsive.

- ***“You always have paperwork to do?***
- ***First I do the paperwork, and there is so much…***
- ***Yeah.***
- ***Payments and everything. It’s a lot of work.***
- ***Yeah. And what about your husband during that time?***
- ***He looks at me.”***

***ON: While his wife is washing, the PA makes phone calls about administrative problems with the bank.***

#### Go through life transitions

The arrival of disease within a family requires several transitions in life – the transition from the role of a spouse / child / friend to that of a caregiver, obviously, but also the different stages of life generated by the pathology of the loved one. Relatives start seeking help from their networks to learn about resources they can use to help their loved ones. As time passes, they become aware of the difficulties of managing and organizing themselves to prepare for the coming difficult stages of life; they realize they need to take care of themselves so they can take care of each other.

To facilitate these transitions in life, caregivers adopt attitudes to better manage the challenges they encounter while accompanying their loved ones. They gradually become aware of their roles as caregivers and are confronted with different stages of difficulties that they must overcome. To do this, they adopt attitudes favouring the passage of the different stages related to the disease. They gain awareness on and ascribe meaning to difficulties, which allows them to put certain situations into perspective and thereby show the resilience to progress towards acceptance of the disease.

- ***“I ... I did not realize that I was at the end of my rope. That’s why now, when I feel that I’m at the end, that I ... uh, when I realize when I ... [Pause] I need to escape, I need ... to ... scream, go out or do something, and then I go out…and then I ... yes, then it’s more pleasant for him, because after I get it out, I am much more calm. I am better ...”***

#### Acquire and transmit knowledge and know-how

To acquire knowledge or know-how and the ability to accompany their close relatives, caregivers adopt self-taught behaviours and continuously stimulate their learning in order to identify essential benchmarks. Caregivers must be open to learning and have self-assessment skills that allow them to adjust their practices.

***ON: To understand the disease, the caregiver will listen to conferences and participate in a support group (strategy for acquiring knowledge).***

#### Protect family dynamics

The arrival of disease in a family requires action by the caregiver to maintain the primary function of the loved one and promote the implementation of his/her new function. Caregivers gradually monitor family dynamics by regularly communicating, for example, the health status of a loved one as well as by resolving family conflicts.

To maintain family dynamics, caregivers seek to promote family exchanges by prioritizing important moments and respecting the rhythm of family members. To do this, they pay attention to the needs of others involved by showing honesty and understanding. Caregivers are in the consensus, transparent and authentic.

- ***“... now that’s what I need ... I’m starting to think a little more about my family. I became aware of this because after – once my mom is gone, and well ... my children will have aged, my grandchildren will grow up and I will say, ‘What did I do for them?’ I have to share myself, huh”.***

#### Protect the health of loved ones

The purpose of accompanying a loved one in his/her daily life, among other things, is to protect that person’s physical and psychological health. Caregivers guarantee the maintenance of their loved ones’ self-esteem, ensuring their comfort, assisting their cognitive functions and coping with emergencies. Caregivers substitute their own abilities for those of loved ones while granting them as much autonomy as possible. To alleviate cognitive disorders due to dementia-type illnesses, they accompany the behaviours of their loved ones with gestures or speech. They perform activities related to the needs of their loved ones and creatively use tips and tricks to ensure their safety and fulfilment through the daily activities of nursing care. In fact, according to an evaluation involving daily observation of the functional and intellectual abilities of a loved one, caregivers execute activities in order to maintain the autonomy of their close relatives. While ensuring the safety of their loved ones, caregivers adapt to their disabilities, promote physical exercise and regularly stimulate them by making social links, among other kind acts. Caregivers also compensate for their loved ones’ loss of capacity by offering care throughout their daily lives, administering treatments and guaranteeing a balanced diet.

To be effective in caring, it is important for caregivers to agree to help. They must be sensitive to the well-being of their loved ones and listen to their needs and personal feelings. Further, they must be able to demonstrate responsiveness, perseverance and calm, as well as to adopt a proactive attitude in search of solutions and a willingness to be a trainer and educator.

- ***“You have prepared his clothes, you say?***
- ***Yes, I always prepare his underpants ... I prepare his clothes that he cannot prepare himself anymore ... otherwise he would wear anything!***
- ***OK.”***

***ON: During the shower, the PA tells him what to do and pays attention to the hot water. The goal is for him to wash himself. The PA allows him to do this (promoting the autonomy of the patient).***

***ON: It’s hot. The PA asks the patient to take off his jacket and closes the shutters and the blinds. She goes out with the patient very early in the morning to avoid the heat (ensuring the safety of the patient).***

***ON: On a morning outing, we go around the block. The mother says, “Charles is going to get your coat; it’s cold outside”. We wait outside while he goes to get his coat. After 2 min, the mother goes to see him and says, “Charles, are you ready?” He is not ready and is looking haggardly. The mother says to Charles, “Look for the green K-way that’s on your bed ... Look for your K-way. It is made with a material like this – look here, it’s like mine ... Look, it’s before your eyes. Take it”. Charles takes it. “That’s fine, you can put it on now”. Charles puts it on and his mom exclaims, “Great, that’s fine”. Charles says, “Super,” with a smile.***

#### Protect your own health

To ensure their roles, caregivers must take care of their own health. They express their emotions and adopt coping strategies to maintain social bonds with friends and allow themselves some respite. To accomplish these ends, caregivers solicit friends and family or seek support from their networks, aiming to share their experiences.

To take care of themselves, caregivers must constantly auto-evaluate themselves so that they can better accept losing control. They prioritize events and show resilience. They are involved in decision-making and take initiative that allows them to accept certain situations. They face the unpredictable and develop a capacity for organization and empowerment. And they learn to be selfish while showing kindness to their loved ones.

- ***“You know, when you want to be effective, you have to say stop.”***

***ON: During the holidays, the PA took a person to look after her husband. She had several treatments to do but would manage to juggle her appointments (plan respite).***

#### Defend the rights/wishes/ideas/interests of the dyad

Some caregivers are engaged at a political level to assert the rights of people in their position. On a daily basis, they support their loved ones and help them defend their rights and interests.

Caregivers gets involved and adopt a proactive attitude. Through empathy for their loved ones, they can act as advocates to ensure their relatives’ well-being.

- ***“I’ll see…. I am very active in the ... the group of caregivers [break] of X (day care).***
- ***Yeah.***
- ***We are considering everyone. We made an association.”***

### Maladaptive behaviours in the care of a person with dementia

In cases where caregivers do not have access to information that can help them better communicate with or react to their loved ones, acquisition of skills is more difficult.

Lack of information can promote behaviours that may be harmful to the health of a loved one. Without the proper knowledge, caregivers may lose patience and overwhelm their loved ones with multiple explanations, anger or raised voices, in which case the sick person will be made to feel devalued.

For the sake of kindness, caregivers may end up speaking down to their loved ones, which only limits their autonomy or even freedom.

Inconsistencies between caregivers’ explanations and their behaviours may exist. For example, when caregivers wish for a loved one to remain independent but persist in dressing and feeding him/her, there is incoherency. Caregivers may end up taking risks to manage certain situations. By attending to some areas alone, caregivers may isolate themselves more and more. When caregivers do not ask for help and moments of respite are not put in place, a feeling of burden may occur, and ultimately fatigue sets in because these people neglect their own health.

- ***“Did you eat well, Mr. C? [Silence].***
- ***Did you hear me? Why don’t you answer? Are you doing this to annoy me? So, how do you want me not to get angry? When I talk to him, I talk to the walls. So ask him, you’re deaf? Do you hear me anymore? What am I doing? So – I get angry. So tell me, why do I get angry?”***
- ***“Since this morning, I noticed that every time your husband takes something in his hands, you take it away from him…***
- ***Yes because he’s going to hurt me. I know the consequences. Or he will pour the coffee on the floor ... it starts with his fingers and he ... he makes ... you’ll see.”***

### Transition process

The results of this study highlight 11 skills used by caregivers in situations encountered on a daily basis. To cope with the role of caregiver, previous transition processes are exploited. Caregivers rely on their personal and professional experience. Their life and illness history allows them to bounce back on the difficulties encountered on a daily basis. The values, interests and preferences of caregivers and their loved one with dementia guide their actions. The personalities of the caregivers are also elements to be taken into consideration because they activate the learning process.

**Fig. 2:**
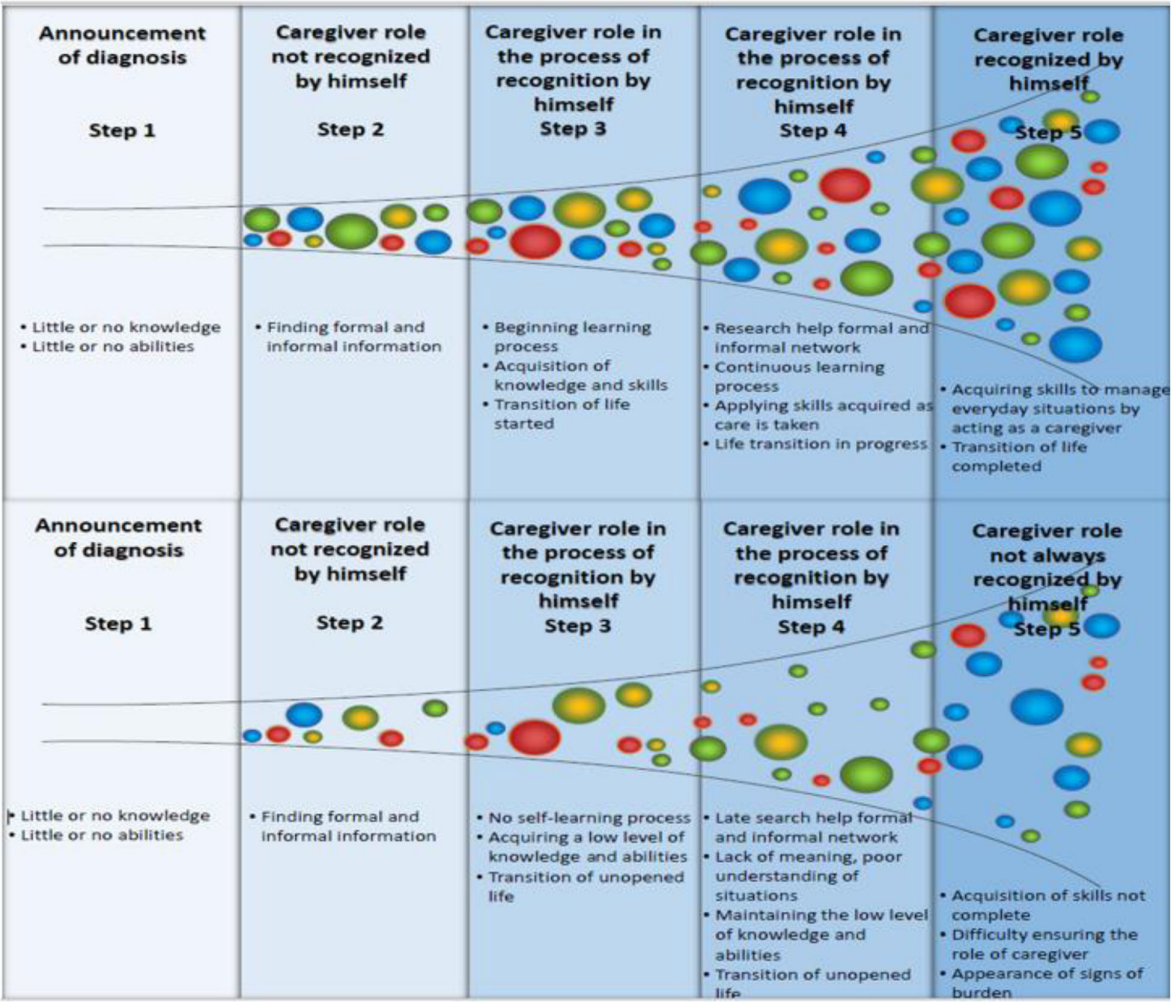
Horizontal process of caregiver life transition and skill development in the case of ability- and knowledge-acquisition in caring and in the case of lack of ability and knowledge-acquisition in caring

In addition, two types of processes stand out (Fig. 2):

- Situations wherein caregivers are undergoing a learning process, which allows them to better understand the context in which they accompany their loved ones. They acquire skills so that they may better handle everyday problems, and the experience of transitioning into a caregiver’s life is facilitated.
- Situations wherein caregivers experience everyday life. They lack knowledge and abilities and do not always understand the context in which they live with their loved ones. They are acquiring skills, but these do not always suffice for the caregivers to live their lives in a more serene way. The experience of transitioning into a caregiver’s life is not easy, and a feeling of burden is often present.

## Discussion

This observational study aimed to identify and characterize the skills of caregivers. The study of the participants’ activities first allowed us to highlight several types of situations encountered daily by caregivers and a number of skills to deal with these situations. Then, in a second step, when the caregivers presented signs related to the feeling of burden, the results made it possible to identify inefficient behaviours in the treatment of the situations.

### The skills of caregivers

Few researchers have studied the skills of caregivers. Farran et al. [18] highlighted seven skills in a study, whereas we through our results were able to put forward 11 skills reflecting the daily lives of caregivers. Some of these skills have developed because the place given to patients and their families is important. Indeed, partnership and care have been evolving from “patient-centric” to “patient–partner models” with patient and family [32, 33]. As stated by Eurocarer [34], “multidisciplinary approaches, including the participation and perspective of family carers, should therefore be supported in order to implement the principles of integrated person-centred care”. This evolution favours a better involvement of patients and their families in the health system and has allowed caregivers to develop their advocacy skills and defend their interests, desires and rights. In addition, because of shifts in ambulatory ability, the involvement of caregivers in caring for their loved ones is even more important. Caregivers develop (as mentioned in this study) skills related to organization, coordination and collaboration, among other traits, with the nursing staff. To ensure that they may continue to accompany their loved ones, they take care of themselves as well, developing skills to maintain their own health and avoid illness. To promote role change and manage the mental load imposed by the patient, acquisition of knowledge and know-how is necessary and entails a learning ability that the majority of participants presented.

Depending on the learning theory, the acquisition of competence is described by different currents. For this study, we discuss learning from the socioconstructivist angle described by Jonnaert [26, 27]. In the literature, caregivers are described as needing, among other things, training and information [35]. The results of our study showed that the majority of caregivers have access to internal, external and specific resources for learning, and that, from a socioconstructivist perspective, caregivers learn to manage everyday situations on their own. As described by Jonnaert’s theory [27], caregivers develop important skills characterized by the combination of knowledge, abilities, use of available resources and adaptation to potential constraints in the care of their loved ones. However, some caregivers lack this self-teaching ability, knowledge and skills, which engenders a lack of meaning – for example, disturbances in the behaviour of sick relatives and potential development of the feeling of burden.

**Fig. 3:**
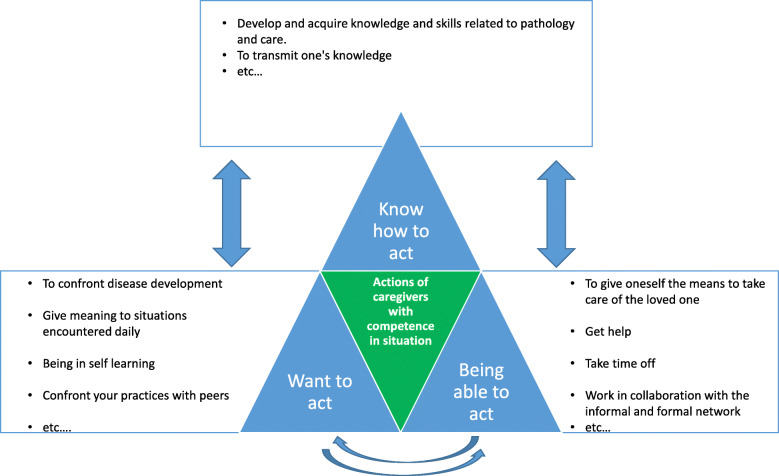
Building the caregivers’ skills. Diagram inspired by Le Boterf, G. Building individual and collective skills, 7th ed. Paris, edition Evrolles; 2015

A model developed by Boterf in the 1990s can be discussed in light of the study results. Boterf indicated that three conditions are necessary to implement a skill: “the conditions related to knowledge (knowing how to act and interact), the conditions related to power (having the power and the means to be able to act and interact) and the conditions related to wanting (having reasons to act and interact)” [36]. In the context of this study, this model is transposable (see Fig. 3). Indeed, some participants did not have the combined triptych of concepts. Some did not know how to act, which prevented them from developing knowledge and skills; others were not psychologically able to act and therefore could not make sense of the different situations they experienced daily; and still others could not act due to a lack of material or human means. Nevertheless, a majority of the participants had all elements of the model, which allowed them to act with relevance and competence in situations.

In addition, the model of competence dynamics, as discussed in the characteristics of different definitions by several authors [27, 36], is specifically present in caregivers of people with dementia. The symptoms of dementia-type conditions accentuate the fact that caregivers must constantly adapt. Indeed, the skills developed 1 day may no longer be valid for the same situation the next day. Despite this, some “isomorphic” situations [27] enable caregivers to accompany people with dementia by resting on the field of their personal experiences. Compared with health professionals, caregivers have greater exposure to the difficulties of taking care of a loved one, so their actions are more specific to the needs of the latter.

Special attention should therefore be paid to self-teaching among caregivers. This approach allows the development of relevant skills, although the risk of basing one’s actions on erroneous information is not zero. Websites from which caregivers learn may sometimes peddle faulty or incomplete information. It is therefore important for family caregivers to have the resources to check their knowledge and prevent it from causing harm to the health of a loved one.

### Maladaptive behaviours for helpers of loved ones

In the context of caregivers, too much mental stress can create a sense of burden, which can facilitate the risk of abuse. Kim et al. [37] characterized the maltreatment risk of people with dementia as “a complex interaction between the characteristics of demented individuals (higher morbidity, severity of cognitive impairment and behavioral and psychological symptoms of dementia) and those of their caregivers (cultural background, psychological morbidity, dysfunctional coping strategy and relationship between the care recipient) and the environment (long-term care, high perceived burden, and low social support)”. This risk may lead caregivers to adopt behaviours harmful to the health of demented relatives. In our study, we observed several behaviours that characterize risks of abuse. In these situations, caregivers are unaware of what they are doing. They find themselves cornered, lacking solutions, and the result is exhaustion. They need the support of outside help, including awareness of the critical situation and searching for a concrete solution to relieve them and bring serenity to the dyad. In such situations, family caregivers struggle to make a life transition experience healthy. In addition, acquiring skills is more difficult for these caregivers because lack of knowledge and sense hinders their progression.

### Limits and interests of the study

The interest of this study is its originality. A majority of qualitative studies on the topic of caregivers are based on methods related to interviews or focus groups. Ours is an observational study, which allowed caregivers’ lives to be identified in a way closer to reality. The researcher was able to visualize the difficulties encountered by caregivers at time T and identify their skills to manage various daily situations alone.

Nevertheless, the study had limitations. A majority of the participants were recruited from the Alzheimer’s Association. Hence, the results may be biased because the participants were already sensitized to the cause and could be seen as more knowledgeable and engaged than isolated caregivers, who would likely be more vulnerable. To overcome this bias, we recruited two caregivers who were not affiliated with any association.

Moreover, an optimal observation would be conducted over several days with the same caregivers. As this was not possible, we designed our study so that different times of the day would be represented. However, despite this precaution, we could not make observations at night. This could have added value to the study because people with cognitive impairment can experience decompensation at night, which generates stress and management difficulties for caregivers.

## Conclusion

In everyday life, caregivers are often alone in their difficulties. They are confronted with new situations throughout the progression of their accompaniment, which obliges them to develop skills for coping with daily life. As they grow in caregiving experience, they progress into new roles, but this learning does not happen simply for all caregivers, and when it is absent, they may be distressed and show signs of fatigue. This study shows that today’s caregivers have developed more skills than their predecessors. This evolution has been accentuated by new paradigms of care requiring caregivers to be more involved. Although some caregivers need training, others may act upon and provide knowledge through their experiences. Partnerships can be envisaged with health institutions so that such actors may better serve their loved ones’ health by their skills.

Emphasizing the competence of family caregivers is an important element because it promotes their legitimacy as participants in the healthcare system. Contrary to the majority of literature related to the activities of family caregivers, such emphasis allows us to note positive elements of caring for sick people at home, which can be valued and recognized not only by direct family members, but also by a caregiver’s entourage.

Finally, identifying the skills of family caregivers and the process of building each skill will better enable caregivers to accompany them, as recommended by Méleis [38].

## Data Availability

The datasets used and/or analysed during the current study are available from the corresponding author on reasonable request.

## Abbreviations

APAF: Association for Assistance and Support for the Elderly in Social Medical Establishments (EMS) and their families
Imad: Institute Geneva Home Support
